# Practical guidance for engaging patients in health research, treatment guidelines and regulatory processes: results of an expert group meeting organized by the World Health Organization (WHO) and the European Society for Clinical and Economic Aspects of Osteoporosis, Osteoarthritis and Musculoskeletal Diseases (ESCEO)

**DOI:** 10.1007/s40520-019-01193-8

**Published:** 2019-04-16

**Authors:** Maarten de Wit, Cyrus Cooper, Peter Tugwell, Nathalie Bere, John Kirwan, Philip G. Conaghan, Charlotte Roberts, Isabelle Aujoulat, Nasser Al-Daghri, Islene Araujo de Carvalho, Mary Barker, Nicola Bedlington, Maria Luisa Brandi, Olivier Bruyère, Nansa Burlet, Philippe Halbout, Mickaël Hiligsmann, Famida Jiwa, John A. Kanis, Andrea Laslop, Wendy Lawrence, Daniel Pinto, Concepción Prieto Yerro, Véronique Rabenda, René Rizzoli, Marieke Scholte-Voshaar, Mila Vlaskovska, Jean-Yves Reginster

**Affiliations:** 1 Department of Medical Humanities, Amsterdam University Medical Centre, Amsterdam, The Netherlands; 2 MRC Lifecourse Epidemiology Unit, University of Southampton, Southampton General Hospital, Southampton, UK; 3 NIHR Musculoskeletal Biomedical Research Unit, University of Oxford, Oxford, UK; 4 WHO Collaborating Centre for Public Health Aspects of Musculoskeletal Health and Aging, Liège, Belgium; 5 Department of Medicine, University of Ottawa, Ottawa, Canada; 6 Public Engagement Department, European Medicines Agency, 30 Churchill Place, Canary Wharf, London, E14 5EU UK; 7 Emeritus Professor of Rheumatic Diseases, University of Bristol, Bristol, UK; 8 Leeds Institute of Rheumatic and Musculoskeletal Medicine, University of Leeds, Leeds, UK; 9 NIHR Leeds Biomedical Research Centre, Leeds, UK; 10 International Consortium for Health Outcomes Measurement, Hamilton House, 4 Mabledon Place, Bloomsbury, London, WC1H 9BB UK; 11 Université Catholique de Louvain, Institute of Health & Society, Brussels, Belgium; 12 Chair for Biomarkers of Chronic Diseases, Biochemistry Department, College of Science, King Saud University, Riyadh, Kingdom of Saudi Arabia; 13 Department of Ageing and Life Course, World Health Organization, 20 Avenue Appia, 1211 Geneva 27, Switzerland; 14 European Patients’ Forum, Chaussée d’Etterbeek 180, Etterbeek, 1040 Brussels, Belgium; 15 Department of Surgery and Translational Medicine, University of Florence, Florence, Italy; 16 Fondazione F.I.R.M.O., Florence, Italy; 17 Department of Public Health, Epidemiology and Health Economics, University of Liège, CHU Sart Tilman B23, 4000 Liège, Belgium; 18 International Osteoporosis Foundation, 9 Rue Juste-Olivier, 1260 Nyon, Switzerland; 19 Department of Health Services Research, CAPHRI Care and Public Health Research Institute, Maastricht University, Maastricht, The Netherlands; 20 Osteoporosis Canada, Toronto, ON Canada; 21 Mary McKillop Health Institute, Australian Catholic University, Melbourne, Australia; 22 Centre for Metabolic Bone Diseases, University of Sheffield Medical School, Sheffield, UK; 23 Scientific Office, Federal Office for Safety in Health Care, Austrian Agency for Health and Food Safety, Vienna, Austria; 24 NIHR Southampton Biomedical Research Centre, University Hospital Southampton NHS Foundation Trust, Southampton, UK; 25 Department of Physical Therapy, College of Health Sciences, Marquette University, Milwaukee, USA; 26 Center for Healthcare Studies, Feinberg School of Medicine, Northwestern University, Chicago, IL USA; 27 Spanish Agency for Drugs and Medical Devices, Calle Campezo 1, Building 8, 28022 Madrid, Spain; 28 Division of Bone Diseases, Geneva University Hospitals and Faculty of Medicine, Geneva, Switzerland; 29 Department of Psychology, Health and Technology, University of Twente, Enschede, The Netherlands; 30 Medical Faculty, Department of Pharmacology, Medical University Sofia, 2, Zdrave Str, 1431 Sofia, Bulgaria

**Keywords:** Outcomes research, Patient engagement, Patient preference, Regulatory process, Treatment guidelines

## Abstract

There is increasing emphasis on patient-centred research to support the development, approval and reimbursement of health interventions that best meet patients’ needs. However, there is currently little guidance on how meaningful patient engagement may be achieved. An expert working group, representing a wide range of stakeholders and disciplines, was convened by the European Society for Clinical and Economic Aspects of Osteoporosis, Osteoarthritis and Musculoskeletal Diseases (ESCEO) and the World Health Organization (WHO). Through a structured, collaborative process the group generated practical guidance to facilitate optimal patient engagement in clinical development and regulatory decisions. Patient engagement is a relational process. The principles outlined in this report were based on lessons learned through applied experience and on an extensive dialogue among the expert participants. This practice guidance forms a starting point from which tailoring of the approach to suit different chronic diseases may be undertaken.

## Introduction

The patient perspective is now increasingly recognized as key in the development, conduct and evaluation of health interventions. The 2015 World Health Organization (WHO) report on healthy ageing identified that self-care plays a critical role in health, with increasing patient participation leading to better health outcomes in older adults including improvements in physical activity, chronic pain and self-efficacy [1]. By involving patients at all stages of healthcare delivery and evaluation, the aim of enhancing self-care in patients with chronic, non-communicable disease may be achieved [2, 3]. Patient-centred care requires an understanding of a variety of patient-relevant components that go beyond medical diagnostic and treatment-related issues. They comprise psychosocial life-related challenges and a focus on patient engagement that acknowledges that patients have an important role to play in their own healthcare. This includes three pillars [4]:

1. *Health literacy* Enabling patient to read, understand and act on quality health information;
2. *Shared decision making and personal care planning* Enhancing self-determination and working with clinicians to select appropriate treatments or management options [5];
3. *Quality improvement* Providing patient relevant feedback on healthcare processes and outcomes.

Patient engagement in healthcare decisions respects patient autonomy increases the likelihood that treatments would be aligned with patient (group) needs and preferences and ultimately leads to better outcomes for all concerned [6]. The complementary impact on society includes reducing healthcare and research waste through the efficient use of resources, as is the focus of health economics [7, 8]. This applies at all levels in society and thus includes individual clinical decisions. The promotion of patient-centeredness is not limited to improving healthcare, but is relevant for the entire life cycle of medicines.

To support the development, approval and reimbursement of medical interventions that best meet patients’ needs, there is increasing emphasis on patient-centred research through the engagement of patients in identifying unmet needs [9], the design and conduct of clinical studies [10], subsequent regulatory assessments [11], post-marketing vigilance and data collection. Patient engagement in clinical practice guideline development is also recommended by many organizations [12–17]. Despite the many ongoing pilots, there is currently little practical guidance on how effective patient engagement may be facilitated. In this paper, we examine how patients’ perspectives can inform the development of clinical research and regulatory decisions. Our aim was to generate practical guidance to facilitate optimal patient engagement in these decisions and to raise awareness of its value.

## Methods

An expert working group meeting was organized by the European Society for Clinical and Economic Aspects of Osteoporosis, Osteoarthritis and Musculoskeletal Diseases (ESCEO) and the WHO, and held in Geneva, Switzerland, on June 9, 2017. The working group comprised a global representation of clinicians, outcome researchers, social scientists, epidemiologists, health technology assessment (HTA) and regulatory experts, pharmacists and patient representatives. They were invited for their expertise and knowledge regarding patient engagement and preference research. Agreement on the principles outlined in this article was based on an exchange of peer-reviewed publications prior to the meeting and a 1 day interactive meeting with short presentations and intense discussion among the meeting participants.

At the meeting, results from a literature review of methods for incorporating patients’ preferences into decision-making were presented followed by an introduction to the nomenclature of patient engagement and a case study on improving the quality of delivering integrated care by involving older people (I. Araujo de Carvalho, I. Aujoulat, M. Hiligsmann). Next, organizations including the European Medicines Agency (EMA) and the Outcomes Measures in Rheumatology (OMERACT) initiative introduced their principles for engagement and presented lessons learned through applied experiences (N. Bere, M. de Wit). After the presentations, a prolonged discussion among the expert group members took place, focused on the application of these principles in different contexts, and shared conclusions were reached.

Following the meeting, members of the writing group (M. de Wit, C. Cooper, N. Bere, P. Tugwell, P. G. Conaghan, C. Roberts, I. Aujoulat) drafted a first report on the meeting, which was reviewed and commented on by all authors. In this resulting article, we define the concepts of patient involvement at different levels and discuss patient engagement within the three areas: patient preference elicitation, outcomes research and regulatory processes. Case studies from the EMA and the Group for Research and Assessment of Psoriasis and Psoriatic Arthritis (GRAPPA) (following OMERACT principles) exemplify our approach.

### Definitions

There are complementary approaches to capturing patients’ perspectives in health research and health services innovation, to ensure that research and development of medical interventions adequately meet patients’ needs. Patients in different numbers may contribute to subsequent phases of a process, carrying out a variety of tasks and on different levels. Different terminology is used, and it is first important to clarify these concepts [18, 19]. We distinguish the following patient roles in patient involvement.

*Consultation* involves participation of patients and/or their representatives as study participants; their role may be to trial an investigational agent and/or to provide information on their own individual experience. This level of involvement requires predominantly a flow of information in one direction from patient to researcher. Protocols for participation in a clinical trial or other means of data collection such as surveys, interviews or focus groups are well developed and highly regulated through the Helsinki declaration.

*Advice* is provided by an informed patient, e.g., by discussing new developments and sharing personal knowledge and experience, or by reviewing research grant applications or a scientific publication. The involvement is often limited to one occasion.

*Collaboration* may be undertaken through multiple roles, e.g., patient research partner (PRP), or patient expert [18]. A patient expert may be defined as an ‘expert’ in their own disease and its management, and someone who is equipped to look further than their own personal experience of the disease. Patient collaboration takes place on a collective level and influences the aims, design and conduct of a study through dialogue, two-way communication in partnership with other stakeholders.

*Control* is achieved when a patient organization takes the lead, determines the research gap or research questions, controls the study and owns the data.

We will use the terms engaging patients and patient involvement as synonyms for the process of enabling people with a long-term condition to provide a meaningful contribution to research or the improvement in healthcare services. Patient empowerment is a process whereby the person develops more control over decisions and actions that affect their own life and health [20, 21]. This involves helping a person to learn to think critically and make informed decisions about self-care to reduce symptoms from the chronic disease [22]. There is increasing recognition that patient empowerment is a core element of quality oriented, sustainable health systems of the future [23]. However, because the focus of this article is on engaging patients in the context of health research and medicine development and authorisation, we will not elaborate on patient empowerment.

## Patient engagement in patient preference elicitation

The assessment of patient preferences could help to improve disease management, facilitate shared decision-making and improve adherence, whilst increasing the use of patient preferences in health policy making may improve the quality of decisions and increase efficiency [24]. For example, the collection of individual preferences may inform regulatory review processes [25]. Preference studies offer a systematic approach to gathering information on the distribution of preferences in a population and the implications on patients’ acceptance of specific treatments. Although the usefulness of stated preference studies is still not well established, such studies, along with other methods such as focus groups and expert opinions, have the potential to become an important tool for gathering patient views in a systematic way to estimate treatment acceptability and concordance, and thus inform regulatory and treatment decisions [26].

Qualitative and quantitative methods could be used to identify what is important to patients with respect to healthcare decision-making. Stated preference methods have been increasingly used in HTA to elicit preference [24]. The most often used stated-preference methods include discrete choice experiment (DCE) [27, 28], best–worst scaling (BWS) [29], and analytical hierarchy process (AHP) [30, 31]. As an example, Rothery et al. used a DCE to assess patient preference for treatment-related benefits and risk of disease relapse in the management of low disease states of psoriatic arthritis (PsA) [32]. The study found that among patients in low disease states of PsA, respondents were willing to accept greater risk of relapse (up to 30% increased risk) for an improvement in the side effects of sickness and nausea and health status.

Patients’ preferences are nowadays increasingly sought in healthcare decision-making. By example, the US Food and Drug Administration (FDA) has developed an initiative for the consideration of patient preferences for multiple endpoints in development assessment of new health technologies in medical devices (FDA Medical Device Patient Preference initiative) [33]. The FDA has also provided definitive guidance on the use of patient reported outcome measures for use in trials to support labelling claims [34], and the EMA has developed guidance on the use of patient reported outcomes in anticancer studies [35].

In Europe, there are several initiatives which aim to increase the use of patient preferences in policy decision-making, which include:

- The German Institute for Quality and Efficiency in Healthcare (IQWiG) may include patient preference in cost–benefit assessment as part of the economic evaluation.
- The National Institute for Health and Care Excellence (NICE) assessment of utility impact involves the elicitation of general population preferences.
- The EMA piloted swing weighting to elicit patient preferences for benefit/risk analysis for market authorization [25]. This was followed up with a larger study eliciting preferences of over 550 multiple myeloma patients on benefits and risks of cancer treatments to illustrate how such data may be used to estimate patients’ acceptance of new treatments [26].
- The PROTECT (Pharmacoepidemiological Research on Outcomes of Therapeutics by a European Consortium) project, conducted by the Innovative Medicines Initiative (IMI) and coordinated by the EMA, focuses on the question of public/patient involvement in regulatory processes [36].
- IMI PREFER (Patient Preferences in Benefit-Risk Assessments during the Drug Life Cycle) project initiated late 2016 focuses on eliciting patient perspectives for use in benefit-risk assessment (BRA) and health technology assessment (HTA) [37]. PREFER will result in recommendations regarding the best ways to perform and include patient-preference in the decision-making process of industry, regulatory authorities and HTA bodies.

These initiatives have focused on the role of patient preferences through the life cycle of medicines and for regulatory decision making. Incorporating the preferences of patients in clinical decision-making (shared decision making) is another dimension that could be the focus of future initiatives [38].

## Patient engagement in clinical outcomes research

Initiatives to increase patient engagement in aspects of clinical research have been undertaken by a wide range of research communities. Engagement may involve the patient, relatives, informal carers or patient representatives, all of whom can give a relevant stakeholder perspective on research and healthcare innovation. The patient engagement process is evolving, and roles for patients may vary with increasing degrees of involvement, from steering group members, committee or working party members with voting rights, to PRPs or Delphi participants [39].

Here we explore the experience of the OMERACT Initiative. OMERACT sets out to achieve consensus about core outcome sets (endpoints) for clinical trials and longitudinal observational studies in rheumatology, with patient involvement an integral part of the process [10]. OMERACT defines the PRP as a person with a relevant disease who operates as an equal member in the research team to provide the patient perspective in every phase of the study [40]. PRPs share decision-making power with professionals, can take on any role similar to all other members and have full voting rights at the biannual meeting [18]. Practical recommendations for patient engagement in research teams were published in 2016 [40].

Other initiatives that have developed rules and pilots to include PRPs in their research activities:

- European League Against Rheumatism (EULAR) published practical recommendations for the inclusion of patient representatives in scientific projects [41]. They are widely followed by all working groups developing disease management recommendations for rheumatic conditions.
- COMET (Core Outcome Measures in Effectiveness Trials) brings together people interested in the development and application of agreed standardised outcome sets used in clinical research [42]. COMET has established a People and Patient Participation, Involvement and Engagement (PoPPIE) Working Group that develops practical resources for PRPs.
- The Standardised Outcomes in Nephrology (SONG) initiative aims to establish a set of core outcomes and outcome measures across the spectrum of kidney disease for trials and other forms of research. The outcomes will be developed based on the shared priorities of patients, caregivers, clinicians, researchers, policy makers and relevant stakeholders. This will help to ensure that research is reporting outcomes that are meaningful and relevant to patients with kidney disease, their family and their clinicians. Currently in development are core outcomes for haemodialysis (SONG-HD), transplantation (SONG-Tx), peritoneal dialysis (SONG-PD) children and adolescents (SONG-Kids), and polycystic kidney disease (SONG-PKD) [43].
- Based on the concept of value-based healthcare, the International Consortium for Health Outcomes Measurement (ICHOM) brings together global teams of physician leaders, outcomes researchers and patient advocates to define Standard Sets of outcomes for clinical practice that matter most to patients. ICHOM captures the patient perspective both by conducting qualitative research to collect data on patient preferences as by active involvement of patient experts in the consensus building process. The final standard sets then drive adoption to enable healthcare providers globally to compare, learn and improve [44].
- The Patient-Centered Outcomes Research Institute (PCORI) has developed the PCORI Engagement Rubric, which provides a framework for operationalizing engagement to incorporate patients and other stakeholders in all phases of research. The aim is to encourage active and sustained engagement of patients as partners in planning, conducting and disseminating research, to improve clinical decision-making and outcomes [45].

### Case study 1: GRAPPA core outcomes set in PsA

GRAPPA is a non-profit organisation focused on identifying and initiating research projects, advancing standardized criteria for PsA registries and developing treatment guidelines. GRAPPA was formed in 2003 of > 600 rheumatologists, dermatologists, radiologists, geneticists, methodologists, epidemiologists and biopharmaceutical industry representatives with an interest in PsA and psoriasis research. Since 2013, patient research partners participate in the annual meetings and a variety of working groups. A best practice case study of patient participation in the update of the PsA core domain set is outlined here [46, 47].

The OMERACT-GRAPPA PsA working group met to review the existing PsA core set [48] based on the patient perspective as well as new research findings and further develop PsA responder indices. The group discussed the need to revise the PsA core set, and opportunities to add, move, or merge existing domains to better integrate the patient perspective and to remove redundancy; and how to incorporate the core set in a composite index [49].

**Fig. 1:**
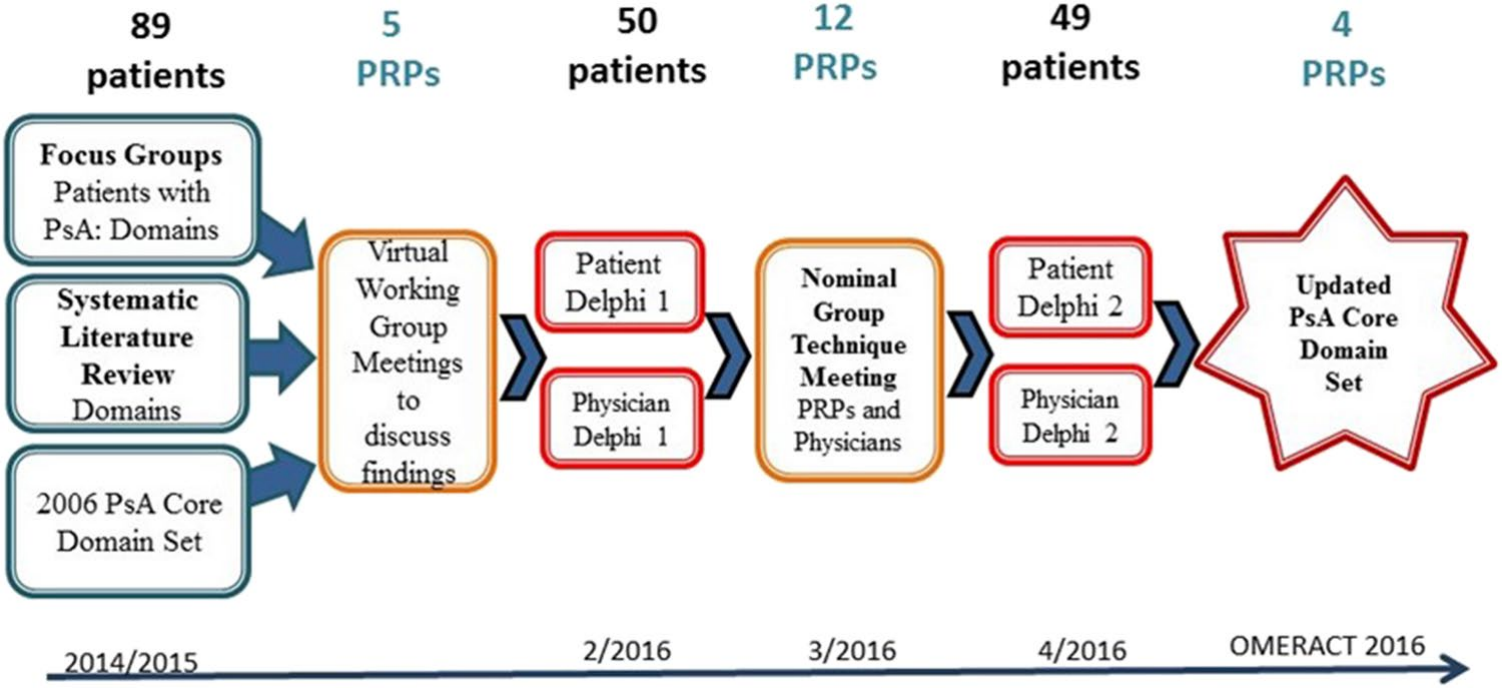
Patient involvement in all steps of the update to the core outcome set for psoriatic arthritis

OMERACT recommendations for patient involvement were followed and included (Fig. 1):

1. Active partnership of five PRPs in the working group and one PRP in the Steering Group.
2. International focus group study representing five continents and including seven countries.
3. Delphi study.
4. Consensus meeting.

New domains were identified through systematic literature review, international focus groups exploring the opinions and experiences of people with PsA and two international surveys with patients and physicians to prioritise domains. Consensus was achieved through an international face-to-face expert meeting with equal representation of patients and physicians to agree on the most important domains, and voting at the OMERACT 2016 conference. PRPs were involved at all phases of the process. As a result, the updated PsA core domain set incorporates patients’ and physicians’ priorities and includes the domains of: musculoskeletal disease activity, skin disease activity, pain, patient global, physical function, health-related quality of life, fatigue and systemic inflammation [47].

#### Lessons learned

Challenges identified were to ensure broad representativeness of patients’ perspectives in demography, geography, disease severity and in numbers, which entails a significant level of communication and efforts to enable equal collaboration. Preserving the patient perspective throughout the research process is of great importance and following from an unanticipated workload for PRPs and researchers it may be difficult to keep PRPs and researchers motivated to collaborate.

Participation requires multiple forms of engagement and should always be tailor-made, there is no common methodology or application that fits every research context. In addition, participation requires a willingness for mutual learning, and the role of the principal investigator is key in providing adequate support to patients. Fruitful participation always requires additional resources to be allocated to the process, with extra effort in time, money and energy. A structural approach guarantees sustainability of participation.

## Patient engagement in regulatory processes

Since establishment of the EMA in 1995, interaction with patients has developed progressively with the creation of a Framework for interaction with patient and consumer groups in 2005, a Patients and Consumers Working Party (PCWP) in 2006, including representatives from 20 organisations and a dedicated ‘public engagement’ department in 2014.

The EMA has increasingly incorporated the patients’ voice within its regulatory activities by way of pilot projects testing the various engagement methodologies, in collaboration with its network of patient organisations [50, 51]. There are now processes to include patients at all stages along the regulatory lifecycle (See Case Study 2).

### Case study 2: EMA methodology on patient engagement, a progressive journey

**Fig. 2:**
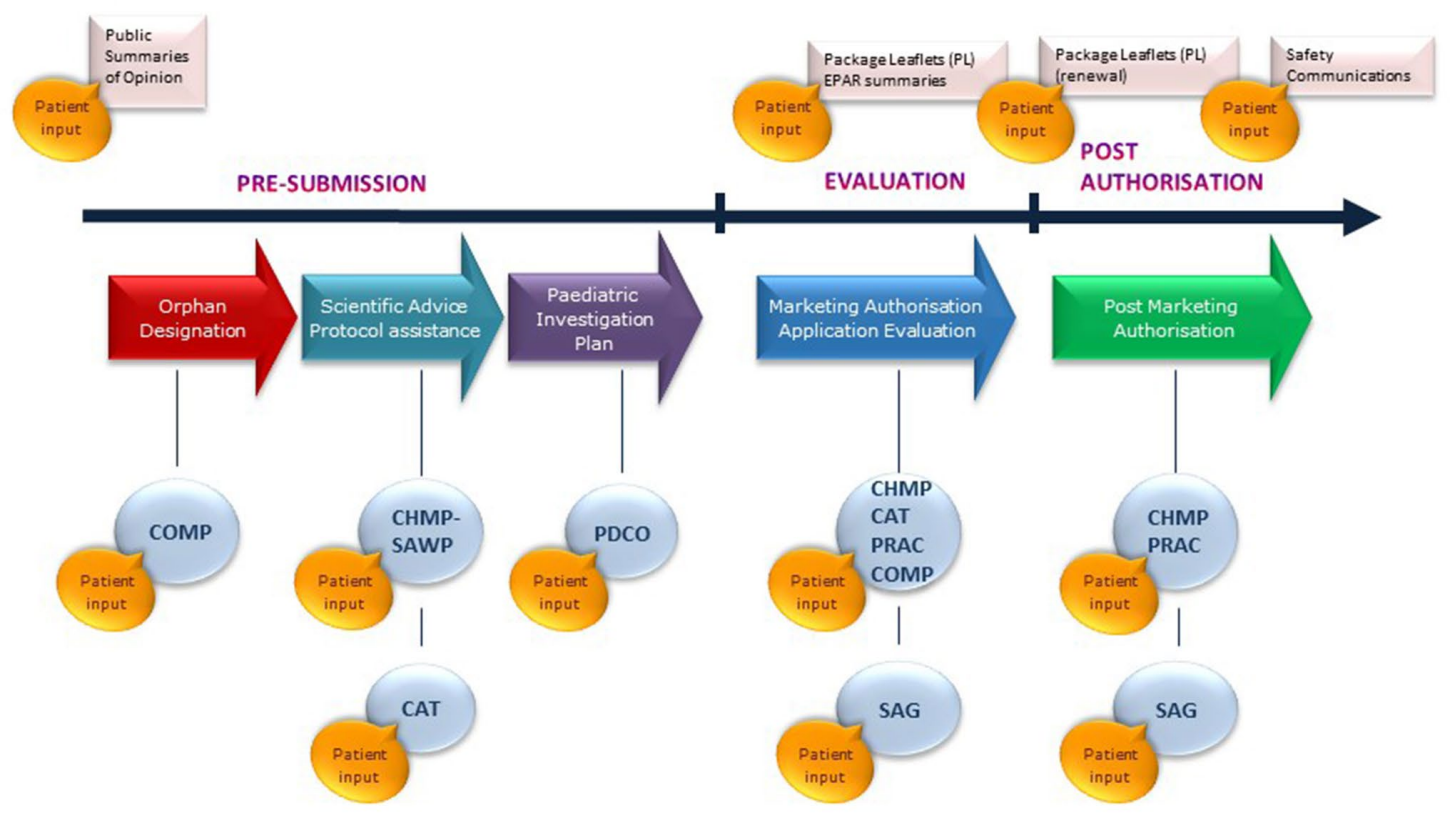
Patient involvement can occur at all steps along the medicine development and regulatory lifecycle

Patient engagement occurs at all steps along the medicine regulatory lifecycle (Fig. 2). As the EMA progressed with involving patients in its activities, it became apparent that there were different levels of patient’s representation, depending on the nature of the activity. The different categories are: representing the patient community; representing their own organisation, and patient as individual experts.

In the pre-submission phase of development, examples of where patients can contribute to EMA discussions on the design of clinical trials include:

- Selection of end-points.
- Defining target population, inclusion/exclusion criteria.
- Study duration, treatment administration, formulation and dosage.
- Clinical relevance versus statistical significance.
- Identification of risk potential.
- Significant benefit (added-value) over existing therapies.
- Ethical aspects, informed consent.

#### Lessons learned

It also became apparent that for optimal and mutually beneficial engagement to occur, the following elements are vital:

- Define the role of the patient for each specific activity and ensure that all involved parties are informed. This facilitates participation and also manages expectations from all angles.
- Identify the most suitable patients/groups to involve in each situation, taking into account representation and confidentiality aspects.
- Establish a range of engagement methodologies and assess for each case which method is most suitable for the situation (e.g., face-to-face, in writing, surveys, committee meetings, preference elicitation).
- Provide individualised support and training, tailored to the individual and the activity, e.g., training sessions, videos, info-sheets, webpages, one-to-one support. To aid training, the European Patients’ Academy on Therapeutic Innovation (EUPATI) has developed patient education resources in key areas of non-clinical and clinical development, clinical trials, regulatory affairs and health technology assessment [52, 53].
- Provide feedback to all involved, especially so that the patient sees that their input is valuable.
- Continuous monitoring and reporting on interactions, and refining procedures according to feedback received.

Challenges identified were in finding suitable patients (e.g., language barrier, availability) while ensuring representativeness and in knowing how to also gather validated information from larger groups. Potential conflicts of interest need to be handled sensitively. Compensation is also an area requiring attention. Demonstrating the value and/or impact of patient involvement in a quantitative manner while avoiding subjectivity is a challenge; however, qualitative methods can be of value provided that there is sufficient reflexivity and transparency. The trend in public health is now to use mixed methods designs.

## Conclusions: principles for engaging with patients

Engaging with patients helps to bridge the gap between health research, policy and patient-centred practice, increases transparency and patients’ adherence to treatments, reduces waste and leads to more meaningful regulatory outcomes. Patient engagement should be initiated in a stepwise approach through which all parties can learn together and identify the format that works best for all involved. At all stages of engagement, provide support, define roles, manage expectations and give feedback, to ensure that engagement is mutually beneficial. In this way, ultimately everyone can benefit from knowledge sharing.

Patient engagement is a relational process, and the nine principles outlined here (see Table 1) [54], form a starting point from which tailoring of the approach to suit different chronic diseases and other healthcare context needs may be undertaken. Overarching principles include the recognition that the perspective of patients is pivotal in outcomes research and the decision-making process of medicine authorisation that earlier involvement of patients is always better and that involvement at all stages is necessary. Patients should be offered the possibility to consult each other on experience-based views; furthermore, patients sometimes need to cancel their presence due to health issues. To ensure proper representation, inviting at least two PRPs is recommended [55]. Finally, acknowledgement of input and feedback to patients is essential, and integrated knowledge translation is desirable [56].

**Table 1:** WHO-ESCEO Principles for engaging patients in health research, treatment guidelines and regulatory processes. Table reprinted from de Wit et al. [54] with permission from Elsevier

9 principles for best practice	
1. Patient perspective	The perspective of patients is pivotal in health research, treatment guidelines, and the authorization of medicines
2. Engagement	Capturing patients’ perspectives requires multiple forms of engagement that are complementary; the strategy should be tailored to suit different chronic diseases and contexts
3. Transparency	Transparency for all stakeholders about the role of patients in the process facilitates participation and manages expectations from all perspectives
4. Representation	Broad representativeness of patients’ perspectives in terms of demography, geography, disease severity and sample size must be ensured
5. Multiple inputs	Involvement of at least two patient experts throughout the research, assessment and deliberation processes ensures that the patient perspective is preserved and increases the validity of the outcomes
6. Support	Providing adequate information, support and feedback to patient representatives is key to effective engagement
7. Expertise	Teaching researchers the knowledge and skills required to support public engagement should always be considered
8. Resources	Productive participation always requires resources to be allocated to the process, with extra effort in time, money and energy
9. Monitor	Continuous monitoring and measuring of interactions will be vital for refining procedures according to feedback

In this article, we acknowledge that there are different levels of patient engagement, all equally valuable and complementary, and that the degree of patient participation and level of power or authority should not be mandatory but may be tailor-made to suit the individual research purpose. Patient engagement is an evolving concept, and the research agenda for future refinement of the process will include the development of new methodologies to assess the impact of patient engagement and novel ways to enhance the significance of existing methods for engagement, in order to emphasize the role of the patient voice in health research, treatment guidelines and regulatory processes. The impact of patient engagement, in terms of added value, but also cost and potential downsides is currently poorly understood. Reasons for this include a lack of consensus on a validated methodology or tool to measure impact, and a lack of consensus on the important outcomes of patient engagement; people and stakeholder groups have different expectations and objectives regarding patient engagement, and thus require different methodologies and outcomes for evaluation.

Another challenge is that we, as an expert group, all agree that principal investigators and stakeholders should invest in support, information, education and feedback to patient experts (see Panel); however, there is a growing awareness that it does not make sense to train patients in the medicine development cycle, without simultaneously preparing researchers for their role and task of engaging patients in that process. Thus, there is a need to explore both the benefits and downsides of educating patient experts as well as exploring the needs of researchers for guidance, coaching and training [57].

## Abbreviations

COMP: Committee for orphan medicinal products
CHMP: Committee for human medicinal products
CAT: Committee for advanced therapies
EPAR: European public assessment report
PDCO: Paediatric committee
SAWP: Scientific advice working party
SAG: Scientific advisory group
PRAC: Pharmacovigilance risk assessment committee
PsA: Psoriatic arthritis
PRP: Patient research partner

## References

[1] WHO (2015) World report on ageing and health. http://www.who.int/ageing/events/world-report-2015-launch/en/. Accessed 24 Jan 2018

[2] Brett J, Staniszewska S, Mockford C (2014). Mapping the impact of patient and public involvement on health and social care research: a systematic review. Health Expect.

[3] Haywood K, Brett J, Salek S (2015). Patient and public engagement in health-related quality of life and patient-reported outcomes research: what is important and why should we care? Findings from the first isoqol patient engagement symposium. Qual Life Res.

[4] Coulter A (2012). Patient engagement–what works?. J Ambul Care Manage.

[5] Coulter A, Entwistle VA, Eccles A (2015). Personalised care planning for adults with chronic or long-term health conditions. Cochrane Database Syst Rev.

[6] Vahdat S, Hamzehgardeshi L, Hessam S (2014). Patient involvement in health care decision making: a review. Iran Red Crescent Med J.

[7] Macleod MR, Michie S, Roberts I (2014). Biomedical research: increasing value, reducing waste. Lancet.

[8] Minogue V, Cooke M, Donskoy A-L (2018). Patient and public involvement in reducing health and care research waste. Res Involv Engagem.

[9] Pittens CACM, Elberse JE, Visse M (2014). Research agendas involving patients: factors that facilitate or impede translation of patients’ perspectives in programming and implementation. Sci Public Policy.

[10] de Wit M, Kirwan JR, Tugwell P (2017). Successful stepwise development of patient research partnership: 14 years’ experience of actions and consequences in outcome measures in rheumatology (omeract). Patient.

[11] Pushparajah D, Geissler J, Westergaard N (2015). Eupati: collaborating between patients, academia and industry to champion the informed patient in medicines research and development. J Med Dev Sci.

[12] Rashid A, Thomas V, Shaw T (2017). Patient and public involvement in the development of healthcare guidance: an overview of current methods and future challenges. Patient.

[13] Montori VMDPJ, Straus S, Haynes B, Guyatt GRD, Meade MO, Cook DJ (2008). Decision making and the patient. Users’ guides to the medical literature: a manual for evidence-based clinical practice.

[14] INVOLVE (2018) The lead for advancement of public involvement in health and care research across nihr and beyond. www.invo.org.uk/about-involve. Accessed 24 Jan 2018

[15] Selva A, Sanabria AJ, Pequeno S (2017). Incorporating patients’ views in guideline development: a systematic review of guidance documents. J Clin Epidemiol.

[16] Diaz Del Campo P, Gracia J, Blasco JA (2011). A strategy for patient involvement in clinical practice guidelines: methodological approaches. BMJ Qual Saf.

[17] Lanza ML, Ericsson A (2000). Consumer contributions in developing clinical practice guidelines. J Nurs Care Qual.

[18] Abma TA, Nierse CJ, Widdershoven GA (2009). Patients as partners in responsive research: methodological notions for collaborations in mixed research teams. Qual Health Res.

[19] PatientPartner (2011) Patient involvement in clinical research. A guide for patient organisations and patient representatives. https://www.geneticalliance.org.uk/media/1602/patientspartnerforpatientorgs.pdf. Accessed 24 Jan 2018

[20] Bravo P, Edwards A, Barr PJ (2015). Conceptualising patient empowerment: a mixed methods study. BMC Health Serv Res.

[21] Barr PJ, Scholl I, Bravo P (2015). Assessment of patient empowerment: a systematic review of measures. PLoS ONE.

[22] Arvidsson S, Bergman S, Arvidsson B (2012). Psychometric properties of the swedish rheumatic disease empowerment scale, swe-res-23. Musculoskeletal Care.

[23] EPF (2016) European patients forum: patient empowerment campaign http://www.eu-patient.eu/campaign/patientsprescribe. Accessed 24 Jan 2018

[24] Bridges JF, Hauber AB, Marshall D (2011). Conjoint analysis applications in health—a checklist: a report of the ispor good research practices for conjoint analysis task force. Value Health.

[25] Postmus D, Mavris M, Hillege HL (2016). Incorporating patient preferences into drug development and regulatory decision making: results from a quantitative pilot study with cancer patients, carers, and regulators. Clin Pharmacol Ther.

[26] Postmus D, Richard S, Bere N (2017). Individual trade-offs between possible benefits and risks of cancer treatments: results from a stated preference study with patients with multiple myeloma. Oncologist.

[27] Clark MD, Determann D, Petrou S (2014). Discrete choice experiments in health economics: a review of the literature. Pharmacoeconomics.

[28] Pinto D, Danilovich MK, Hansen P (2017). Qualitative development of a discrete choice experiment for physical activity interventions to improve knee osteoarthritis. Arch Phys Med Rehabil.

[29] Cheung KL, Wijnen BF, Hollin IL (2016). Using best-worst scaling to investigate preferences in health care. Pharmacoeconomics.

[30] Marsh K, Caro JJ, Hamed A (2017). Amplifying each patient’s voice: a systematic review of multi-criteria decision analyses involving patients. Appl Health Econ Health Policy.

[31] Hiligsmann M, Dellaert BG, Dirksen CD (2017). Patients’ preferences for anti-osteoporosis drug treatment: a cross-european discrete choice experiment. Rheumatology.

[32] Rothery C, Bojke L, Richardson G (2016). A discrete choice experiment to explore patients’ willingness to risk disease relapse from treatment withdrawal in psoriatic arthritis. Clin Rheumatol.

[33] Basch E (2013). Toward patient-centered drug development in oncology. N Engl J Med.

[34] FDA (2009) Guidance for industry: patient-reported outcome measures: use in medical product development to support labeling claims. Clinical/medical 2009 http://www.fda.gov/downloads/drugs/guidancecomplianceregulatoryinformation/guidances/ucm193282.pdf. Accessed 24 Jan 2018 10.1186/1477-7525-4-79 PMC1629006 17034633

[35] EMA (2016) Appendix 2 to the guideline on the evaluation of anticancer medicinal products in man: the use of patient-reported outcome (pro) measures in oncology studies. http://www.ema.europa.eu/docs/en_gb/document_library/other/2016/04/wc500205159.pdf. Accessed 12 Feb 2018

[36] PROTECT (2018) The pharmacoepidemiological research on outcomes of therapeutics by a european consortium. http://www.imi-protect.eu/index.shtml. Accessed 24 Jan 2018

[37] PREFER (2016) Patient preferences in benefit-risk assessments during the drug life cycle. www.imi-prefer.eu Accessed 24 Jan 2018

[38] Coulter A, Collins A (2011) Making shared decision-making a reality: no decision about me, without me. The King’s Fund, London. https://www.kingsfund.org.uk/sites/default/files/making-shared-decision-making-a-reality-paper-angela-coulter-alf-collins-july-2011_0.pdf. Accessed 24 Jan 2018

[39] de Wit MP, Kvien TK, Gossec L (2015). Patient participation as an integral part of patient-reported outcomes development ensures the representation of the patient voice: a case study from the field of rheumatology. RMD Open.

[40] Cheung PP, de Wit M, Bingham CO (2014). Recommendations for the involvement of patient research partners (prp) in omeract working groups. A report from the omeract 2014 working group on prp. J Rheumatol.

[41] de Wit MP, Berlo SE, Aanerud GJ (2011). European league against rheumatism recommendations for the inclusion of patient representatives in scientific projects. Ann Rheum Dis.

[42] Williamson PR, Altman DG, Bagley H (2017). The comet handbook: version 1.0. Trials.

[43] SONG (2018) The standardised outcomes in nephrology (song) initiative: an international initiative that aims to establish core outcomes in chronic kidney disease. http://songinitiative.org/ Accessed 24 Jan 2018

[44] ICHOM (2018) International consortium for health outcomes measurement. www.ichom.org. Accessed 24 Jan 2018

[45] Sheridan S, Schrandt S, Forsythe L (2017). The pcori engagement rubric: Promising practices for partnering in research. Ann Fam Med.

[46] Tillett W, Adebajo A, Brooke M (2014). Patient involvement in outcome measures for psoriatic arthritis. Curr Rheumatol Rep.

[47] Orbai AM, de Wit M, Mease P (2017). International patient and physician consensus on a psoriatic arthritis core outcome set for clinical trials. Ann Rheum Dis.

[48] Gladman DD, Mease PJ, Strand V (2007). Consensus on a core set of domains for psoriatic arthritis. J Rheumatol.

[49] Tillett W, Eder L, Goel N (2015). Enhanced patient involvement and the need to revise the core set—report from the psoriatic arthritis working group at omeract 2014. J Rheumatol.

[50] EMA (2011) Outcome report on pilot phase for participation of patient representatives in scientific advisory group (sag) meetings. http://www.ema.europa.eu/docs/en_gb/document_library/report/2011/12/wc500119201.pdf. Accessed 24 Jan 2018

[51] EMA (2017) Outcome report on pilot to involve patients in benefit/risk discussions at chmp meetings. http://www.ema.europa.eu/docs/en_gb/document_library/report/2017/05/wc500227335.pdf. Accessed 24 Jan 2018

[52] EUPATI (2018) European patients’ academy on therapeutic innovation (eupati) https://www.eupati.eu/. Accessed 24 Jan 2018

[53] Chakradhar S (2015). Insurance companies are slow to cover next-generation sequencing. Nat Med.

[54] de Wit MC, Reginster J-Y (2019) Practical guidance for patient-centred health research. Lancet 393:1095–1096. 10.1016/S0140-6736(19)30034-0 10.1016/S0140-6736(19)30034-0 30894262

[55] Hewlett S, Wit M, Richards P (2006). Patients and professionals as research partners: challenges, practicalities, and benefits. Arthritis Rheum.

[56] Tunis SR, Maxwell LJ, Graham ID (2017). Engaging stakeholders and promoting uptake of omeract core outcome instrument sets. J Rheumatol.

[57] de Wit M, Beurskens A, Piskur B (2018). Preparing researchers for patient and public involvement in scientific research: development of a hands-on learning approach through action research. Health Expect.

